# Obesity and Diabetes Mediated Chronic Inflammation: A Potential Biomarker in Alzheimer’s Disease

**DOI:** 10.3390/jpm10020042

**Published:** 2020-05-22

**Authors:** Md Shahjalal Hossain Khan, Vijay Hegde

**Affiliations:** Obesity and Metabolic Health Laboratory, Department of Nutritional Sciences, Texas Tech University, Lubbock, TX 79409, USA; Md-Shahjalal.Khan@ttu.edu

**Keywords:** obesity, diabetes, inflammation, Alzheimer’s disease, Amyloid Beta, Tau, biomarker, mitochondrial dysfunction

## Abstract

Alzheimer’s disease (AD) is the sixth leading cause of death and is correlated with obesity, which is the second leading cause of preventable diseases in the United States. Obesity, diabetes, and AD share several common features, and inflammation emerges as the central link. High-calorie intake, elevated free fatty acids, and impaired endocrine function leads to insulin resistance and systemic inflammation. Systemic inflammation triggers neuro-inflammation, which eventually hinders the metabolic and regulatory function of the brain mitochondria leading to neuronal damage and subsequent AD-related cognitive decline. As an early event in the pathogenesis of AD, chronic inflammation could be considered as a potential biomarker in the treatment strategies for AD.

## 1. Introduction

Alzheimer’s disease (AD) is a progressive and irreversible brain disorder that begins well before the clinical symptoms appear. The actual symptoms may appear only after several years of changes in the brain due to damage or destruction of brain cells (neurons) in the area involved in cognitive function such as memory, thinking, and learning [1]. AD slowly abolishes brain function and hinders thinking ability. AD is recognized as a common cause of an estimated 60–80% of cases of dementia [2]. The early clinical symptoms of AD have been described as difficulty in conversations and depression and is followed by disorientation, confusion, impaired communication, behavioral changes, and, eventually, trouble in speaking and walking. At the critical stage, consequences are fatal and the patients are bed-bound and need special attention such as round-the-clock care [3].

AD prevalence is escalating rapidly worldwide especially among the population aged 65 years and older. In the United States, it has been reported that, in 2019, an estimated 5.8 million Americans were living with AD-related dementia and, among them, 81% were aged 75 or older and 200,000 individuals were under 65 years of age [3]. The numbers have been projected to grow from the current 55 million to 88 million [3], approximately doubling by 2050 [4]. This alone depicts the magnitude of the burden of AD on health care and the overall society in the future. Considering disability-adjusted life years (DALYs) as a primary measure of disease burden, in the United States, AD has risen from the 12th most burdensome disease in 1990 to the 6th most burdensome disease in 2016 [5]. It has been estimated that, in 2019, the healthcare burden will be around $290 million [3]. Collectively, the above statistics indicate that AD does not only affect morbidity or mortality, but AD is also affecting the socio-economic and healthcare burden in the USA. There have been numerous studies elucidating the pathogenesis, molecular and clinical mechanisms, the association of diabetes and metabolic syndromes, and consequences in a broader range. Yet no single or combined treatment has shown to have satisfactory levels of efficiency to delay or prevent AD pathogenesis.

In this review, we will focus on a much narrower context of AD pathogenesis considering inflammation as the central mechanistic link among obesity, diabetes, and AD-related dementia. In obesity, the elevated circulating free fatty acids (FFA) attributes to inflammation, initiated by Toll-like receptors (TLR-4) and release of pro-inflammatory cytokines, which is also associated with insulin resistance and diabetes [6,7] via nuclear factor-kappa B (NF-*k*B) mediated inflammation that might mediate the intracellular signaling impairments [8]. In the brain, pro-inflammatory cytokines activated by microglia can induce oxidative stress and compromised antioxidant defense [9]. Collectively, they contribute to impaired insulin signaling, synapses loss, reduced mitochondrial axonal transport [10], mitochondrial fragmentation, dynamics, and eventual dysfunction [11]. Mitochondrial dysfunction has been considered as an early event for AD pathogenesis and is also associated with a metabolic syndrome including obesity, diabetes, insulin resistance, and cardiovascular diseases [8]. Thus, it is possible that mitochondrial dysfunction has an evident role in the initiation and development of the metabolic disorder, and it is worthy to investigate to what extent inflammation and mitochondrial health affect the progression of AD.

## 2. Pathogenesis of AD

The characteristic features of AD involve two major fundamental processes: extracellular beta amyloid (A*β*) deposition and intracellular tau protein hyper accumulation. A*β* is insoluble and is a major component for senile plaques. Insoluble tau is the major component of neurofibrillary tangles (NFT) [12]. A*β* is a 36 to 43 amino acid peptide, which is a part of the large transmembrane protein, Amyloid Precursor Protein (APP), and derived from cleavage of APP by *β*- and *γ*–secretase enzymes. Defective clearance of A*β* during the cleaving process of APP results in the accumulation of insoluble A*β* [13]. Initially, the A*β* monomer polymerizes into soluble oligomers and then into larger insoluble fragments like A*β*42 that can precipitate as amyloid fibrils [14]. On the other hand, tau is a protein associated with the microtubule and helps in modulating the axonal microtubule stability [15]. In an AD patient’s brain, the tau protein gets hyper-phosphorylated and causes the protein to lose microtubule-binding ability and dissociate from the microtubules, which can progressively disrupt the transport structure and result in starvation of neurons and, ultimately, neuronal cell death [16]. Deposition of neuronal bodies and processing of insoluble phosphorylated tau protein into paired helical filaments can cause neurofibrillary degeneration. These deposits interfere with the spacing between microtubules and hinder the axonal terminals and dendrite nutrient transport [17].

## 3. Obesity, Diabetes, and AD

Obesity is a chronic and multifactorial disease characterized by excessive body fat accumulation. It is considered a risk factor for many diseases and disorders including hypertension, type 2 diabetes mellitus, coronary heart disease, and AD [18]. Adipose tissue possesses endocrine function secreting adipokines, inflammatory cytokines, and other bioactive mediators that influence energy homeostasis in metabolically active organs like adipose tissue, liver, pancreas, and even brain [19]. During a positive energy balance, adipocyte size and proliferation increases result in the expansion of adipose tissue to accommodate the excess energy, which leads to an increase in adipose tissue mass. This is followed by adipose tissue dysfunction promoting chronic low-grade inflammation [20], elevated oxidative stress [21,22], and altered mitochondrial dysfunction [23,24,25]. Although how obesity contributes to the mitochondrial dysfunction is still not clear, it is postulated that the induced inflammation and metabolic alteration implicated by impaired insulin function could be a possible factor [26,27].

Altered glucose homeostasis due to hyperinsulinemia and insulin resistance in diabetes is also one of the most prominent features of obesity. Obesity and a high fat diet (HFD) can induce insulin resistance, which, subsequently, impairs insulin signaling in the periphery as well as in the brain [28]. It is well-established that insulin has a significant role in the central nervous system (CNS) [29,30,31,32,33]. In the brain, insulin along with insulin growth factors (IGF) modulate neuronal growth, differentiation, survival, migration, metabolism, protein synthesis, gene expression, synapse formation, and synaptic plasticity [34]. Moreover, insulin regulates myelin production and oligodendrocyte maintenance [34]. Defective insulin signaling is also associated with impaired cognition and AD-related dementia [35]. Severely decreased phosphorylation of insulin receptors has been found in patient brains from both AD and diabetes [36]. The disturbance in insulin signaling could contribute to making the CNS environment more vulnerable to metabolic stress and, therefore, accelerate the neuronal dysfunction [37]. Diabetes is associated with islet amyloid polypeptide (IAPP) accumulation in the pancreatic islets. The IAPP is concurrently secreted with insulin from the pancreas. In diabetic and AD patients, the IAPP is found to be misfolded and elevated [38], accompanied by elevated A*β* accumulation, hyper-phosphorylated tau, and impaired fasting glucose as comorbidities [39].

Substantial evidence indicates that patients with obesity and diabetes are more susceptible to develop AD-related cognitive degeneration. Several longitudinal [40,41] and epidemiological studies [42,43,44,45] have established a significant association between midlife large waist-hip ratios to AD-related dementia and decreased hippocampal volume in later life. Moreover, the common pathways of neurodegeneration in the AD brain resemble similar pathologies observed in the brains of individuals with diabetes. Obesity, AD, and diabetes concomitantly share common features like brain atrophy, reduced cerebral glucose, and CNS insulin resistance [46].

## 4. Obesity, Diabetes, and Subsequent Systemic and Neuro-Inflammation

Obesity, diabetes, and AD attributes to a common shared chronic inflammatory process. Both epidemiological and observational studies suggested that neuro-inflammation is an early-stage marker of AD pathogenesis [47]. A higher plasma and CNS levels of inflammatory markers known as Interleukin-6 (IL-6), Interleukin 1*β* (IL-1*β*), transforming growth factor-*β* (TGF-*β*), and tumor necrosis factor-*α* (TNF-*α*) has been reported in AD patients, which indicates the potential role of inflammatory markers in AD pathogenesis [48]. Insulin resistance and inflammation have a bidirectional relation with AD. In the chronic peripheral inflammatory process in obesity and diabetes, the production of inflammatory cytokines can lead to serine phosphorylation of insulin receptor substrate-1 (IRS-1), which can inhibit the downstream signaling pathways like kappa B kinase (IKK), c-Jun N-terminal kinase (JNK), and extracellular signal regulated kinase 2 (ERK2). These can block the intracellular insulin signaling by downregulating the insulin receptor-mediated signaling [49]. Additionally, the systemic inflammation can damage and cross the blood-brain barrier (BBB) and enter into the brain, which might trigger the brain-specific inflammatory response [50]. These circulating cytokines can increase the apoptosis (cell death) rate, reduce synaptic function, and inhibit the neurogenesis and, thus, causing neuronal death [51]. Moreover, systemic inflammatory processes, e.g., IL-6, TNF-*α*, and C-reactive proteins (CRP), inhibit the transfer of A*β* from the CNS to the periphery [52]. Thus, A*β* oligomers accumulation can activate brain microglia that secrete the pro-inflammatory cytokines (IL-1*β*, IL-6, TNF-*α*), which can phosphorylate insulin receptor substrate (IRS) in multiple sites by activating the IRS-1 serine kinase upon binding with the respective receptors and alter brain insulin signaling [53]. Although the precise mechanisms are yet to be understood, the putative mechanisms for systemic and neuro-inflammation caused by obesity and diabetes are as follows.

### 4.1. Systemic Inflammation and AD

The circulating pro-inflammatory molecules either increase the permeability of the BBB or gain access via the area that lacks effective BBB to initiate neuro-inflammation in the hypothalamus [54,55]. The mechanism of BBB disruption is multifaceted and includes changes in the structural components like pericyte dysfunction, tight junction, and elevated endothelial oxidative stress [56]. HFD can elevate the expression and activation of pro-inflammatory cytokines and corresponding transcription factors, such as NF-*k*B, in the hypothalamus [57]. This pro-inflammatory action (a) will increase the microglial (brain’s residing macrophage) infiltration and activation in the hypothalamus resulting in the local inflammatory mediators such as cytokines [57,58]. In addition, the elevated free fatty acids (FFA) enters the arcuate nucleus and increases TLR-4, a molecular pattern recognition receptor, on microglia and astrocytes and initiates the inflammatory response centrally [59]. (b) The circulating cytokines have limited spread and activate the hypothalamic cytokine receptors. This action can augment the brain inflammation [28]. (c) The direct entry of cytokines, chemokines, and FFA in systemic circulation can also propagate neuro-inflammation by initiating pro-inflammatory cytokines and prostaglandin production [60]. Collectively, this pro-inflammatory milieu can disrupt the hypothalamic function by inducing synaptic remodeling, neuronal cell apoptosis, and disturbed neurogenesis [61]. The hypothalamus has a potential role in cognitive function related to feeding, metabolism, stress regulation, and cardiovascular function along with cognition, attention, and memory function [62]. The remodeling of the hypothalamic circuit leads to dysregulation in the hypothalamic-pituitary-adrenal (HPA) axis resulting in increased production of glucocorticoids, which are related to impaired cognition and memory including depression, Cushing’s syndrome, and AD [63]. Moreover, the chronic HPA axis activation and elevated glucocorticoid level is also associated with dendritic atrophy, hippocampal volume reduction, and reduced synaptic plasticity [64]. One of the characteristic features of AD is the hippocampus and cerebral cortex atrophy [65]. All these possible events collectively can lead to neurodegeneration and eventually AD.

### 4.2. Neuro-Inflammation in AD

The brain was thought to be unaffected by systemic inflammation and, thus, regarded as an “immune-privileged” organ not susceptible to inflammation for many years. However, this notion changed after extensive neuro-immune research that explored the central nervous system interaction with the peripheral system through hormonal and paracrine action [66]. In the case of the AD brain, A*β* deposition triggered by systemic inflammation initiates a series of immune-responses intended to reduce the A*β* plaques and the aggregates by activating the innate immunity to elicit the inflammatory response from microglia and astrocytes [67,68]. These pathological adaptations incite the release of pro-inflammatory cytokines including IL-6, IL-1*β*, or TNF-*α* along with other pro-inflammatory molecules including macrophage inflammatory proteins, monocyte chemoattractant proteins, coagulating factors, reactive oxygen species (ROS), nitric oxide, proteases, protease inhibitors, prostaglandins, thromboxanes, leukotrienes, and CRP from glial cells [52,69,70,71]. The aggravated environment induces additional phosphorylation of tau, accumulation of A*β*, and pro-inflammatory molecules [52], which, consequently, release reactive substances like nitric oxide, proteolytic enzymes, excitatory amino acids, and complementary factors, and damage the adjacent healthy neurons [72]. Therefore, the well-intended initial response to assist A*β* clearance, in turn, also secretes mediators that cause damage and leads to neurodegeneration [73]. An elevated level of IL-1*β* was found in serum, cerebrospinal fluids, and in the brain region [74,75], particularly astrocytes of the cortex and hippocampus [76] of patients with AD. The IL-1*β* secreted from astrocytes increases the neurotoxic A*β* and APP production [76,77]. The IL-1*β* also activates microglia to induce pro-inflammatory cytokine release that can lead to neurotoxicity [78]. Additionally, oxidative damage and alteration in tau phosphorylation in neurons, along with chronic low-grade inflammatory states, collectively lead to compromised brain integrity [79].

### 4.3. RAGE-Mediated Inflammation in AD

Dyslipidemia and chronic hyperglycemia caused by disturbance of insulin signaling in obesity can lead to glucolipotoxicity, which can play a pivotal role in AD. Hyperglycemia also generates advanced glycation end products (AGE), which is a senescent macro-protein derivative that can be identified in both senile plaques and NFTs as a possible link between AD and diabetes. AGEs are the derivatives of proteins, lipids, or nucleic acids modified by non-enzymatic glycosylation and tend to increase the production and accumulation during aging, diabetes, or obesity [80,81]. Advanced glycation end products (AGE) and its pattern recognition receptors (RAGE) induce a chronic, persistent inflammatory response that propagates vascular injuries [81,82,83]. RAGE is also a putative receptor for A*β* [84] and is found to be expressed in neurons, microglia, and astrocytes [85,86]. A*β* is a ligand for RAGE that interacts with the N-terminal domain. Neurons and microglia of the hippocampus and inferior frontal cortex area of the AD brain have reported expressing an elevated level of RAGE [85,87].

The A*β*-RAGE interaction in the neuronal cells leads to an ROS mediated cellular response and activation of NF-*k*B that induce elevated inflammatory milieu [87]. RAGE dependent A*β*-induced migration has been shown in both in-vitro A*β* plaque models and postmortem human microglia cell culture experiments [85]. It was reported that A*β*-RAGE dependent microglial activation elevates the RAGE and microglial colony-stimulating factors (M-CSF), while inhibition of RAGE using anti-RAGE F(ab′)_2_ inhibits the microglial chemotactic response against A*β*_42_ [85]. Moreover, increased production of IL-1*β* or TNF-*α*, enhanced microglial infiltration in A*β* plaques, reduced acetylcholine esterase activity, and impaired spatial and learning memory observed in double transgenic mouse models when compared to APP or RAGE mouse models [88]. Elevated levels of AGE in the brain and plasma are associated with the cognitive dysfunction in AD patients [89,90]. AGE, along with increasing A*β* cytotoxicity, supports fibrillary tangles and A*β* plaque formation, which contributes to the pathogenesis of AD [91].

## 5. Inflammation Mediated Impaired Mitochondrial Health in AD

Mitochondria, which is the powerhouse of cells that provides energy by utilizing ATP, is also responsible for cellular processes including energy metabolism, ROS generation, calcium ion (Ca^2+^) homeostasis, cell survival, and apoptosis [92]. Mitochondria maintain the normal functioning through regulated quality control even in the presence of persistent insults. However, any failure of quality control results in mechanical defects and damages that cause mitochondrial dysfunction [93]. It can lead to metabolic disorders. The major metabolic organs like liver, muscle, and adipose tissue are the active contributors in this process. During obesity and diabetes, excess lipid accumulation and hyperglycemia induced insulin resistance can lead to abnormal mitochondrial function via altering the ATP synthesis activity of mitochondria, beta-oxidation, and elevated oxidative stress [94]. Adipocyte differentiation drastically increases the activity and biogenesis of mitochondria, pointing towards a link between mitochondria and obesity. Mitochondrial dysfunction is correlated with a reduced size and number of mitochondria [95], oxidative capacity [96], reduced oxygen consumption, and oxidative phosphorylation related gene expression [97], which have been observed in individuals with obesity [90]. In mature adipocytes, mitochondrial dysfunction has been linked to fatty acid oxidation [98], adipokine secretion [99], and impaired glucose homeostasis [100]. Additionally, in obese mice, the expression of mitochondrial DNA (mtDNA), mtDNA transcription factor A (Tfam), and respiratory proteins are also significantly reduced [92]. HFD increases the production of ROS and oxidative stress in mouse adipocytes [101]. HFD consumption or excess calorie intake results in an excess mitochondrial load that hinders the effective dissipation of the proton gradient, which increases ROS generation, mtDNA mutation, and apoptosis [102].

There are several ways obesity and diabetes-induced inflammation affects mitochondrial functioning.

### 5.1. Inflammation and Energy Metabolism

Neuronal glucose metabolism consists of mechanisms that regulate uptake of glucose, insulin, and insulin signaling pathways, glucose transporters (GLUTs), and entry of glycolytic end-products into mitochondria that eventually metabolize and generate ATP via oxidative phosphorylation [103]. Mitochondria participates and modulates several metabolic signaling pathways including cytosolic signaling, redox-sensitive signaling, JNK, and 5′ AMP-activated protein kinase (AMPK) signaling [104]. These signaling pathways along with the associated metabolites, transporters, receptors, and enzymes ensure proper neuronal energy metabolism. Mitochondria-centered altered glucose metabolism manifested by impaired insulin signaling, altered receptors activity, and reduced glucose uptake is one of the key features of AD [104]. Mitochondria regulate the tricarboxylic acid (TCA) cycle, which is the principal regulator of cellular respiration. The TCA cycle generates adenosine triphosphate (ATP) via a series of enzyme-catalyzed chemical reactions and any malfunction in the enzymes involved in this cycle leads to impaired cellular respiration and ATP production [104].

Inflammatory processes mediated by infiltrated immune cells from the periphery and activated microglia initiate an intracellular signaling cascade that modifies the mitochondrial energy metabolism [105]. The activated microglia and astrocytes release pro-inflammatory cytokines, particularly TNF-*α*, and induce oxidative phosphorylation impairment, ATP production, and ROS production [105]. In one study, loss of mitochondrial transmembrane potential and reduction in intracellular ATP upon treatment with IL-1*β* in the retinal neuronal cells has also been reported [106]. Moreover, IL-1*β* and TNF-*α* are reported in decreasing the pyruvate dehydrogenase (PDH) enzyme activity in vivo [107], particularly in cardiomyocytes [108]. Reduced glucose oxidation in cultured human dermal fibroblast [109] reduced skeletal muscle [110,111], hepatic PDH activity [107], and complex I and II activity reported in the presence of inflammatory cytokines [105]. Impaired PDH activity has been found in the postmortem AD brain along with increased IL-6, IL-1*β*, or TNF-*α,* which suggests a compromised TCA cycle activity in the inflamed CNS of mild cognitive impairment and AD patients [105]. A significant reduction of neuronal complex I and III along with several other nuclear-encoded subunits of the Electron Transport Chain (ETC) has also been reported [112]. A reduction of ATP production and basal respiration upon TNF-*α* exposure has been shown in a dose-dependent manner in the mouse hippocampal cell line and primary neuronal cell culture [113]. TNF-*α* administration also reduces the peroxisome proliferator-activated receptor (PPAR)-γ co-activator 1*α* (PGC-1*α*) in myoblasts [114] and human cardiomyocytes [115] even though the effect of TNF-*α* on neuronal cells of various neurodegenerative diseases has not yet been reported. PGC-1*α* plays an important role in many cellular and metabolic processes including energy metabolism, cardiovascular diseases, and neurodegenerative diseases [116].

It has been reported that, in the AD brain, the levels of mitochondrial enzymes including PDH, cytochrome oxidase (COX), and *α*-ketoglutarate dehydrogenase complex are all decreased [117]. Proteomics studies on AD brains reveal that enzymes involved in metabolic pathways of the Kreb’s cycle and glycolysis including malate dehydrogenase, glyceraldehyde 3-phosphate dehydrogenase (GAPDH), fructose-bis-phosphate-enolase, alpha-enolase (ENO1), and ATP synthase are oxidized [46]. The oxidation of these enzymes is related to dysfunctional cerebral glucose metabolism and reduction in ATP synthesis, which leads to loss of synaptic function [118]. These oxidative changes can lead to inflammation and eventually compromise metabolic function. The exact mechanism of how neuro-inflammation affects the metabolic function of mitochondria is yet to be understood. However, cellular and biochemical studies revealed that APP, A*β*, tau, and presenilin are associated with impaired mitochondrial energy metabolism [119,120]. It has been proposed that A*β* interacts with mitochondrial proteins disrupting ETC, increases ROS production, and free superoxide radicals that preclude the cellular ATP generation [121]. It has also been proposed that the hyper-phosphorylated tau and A*β* aggregates can block mitochondria and other cell organelles from nerve terminals, synapsis, and other brain regions with high ATP demands [122], which might lead to starvation of dendritic spines and synapsis due to severe mitochondrial ATP depletion [123].

### 5.2. Inflammation and Altered Mitochondrial Dynamics

Mitochondria are organelles that exist in dynamic networks, migrate throughout the cell, continuously fuse, divide, and undergo regulated turnover as the metabolic environment demands [124]. Mitochondrial dynamics can change the size and shape and adapt to the challenges [125]. Mitochondrial fission and fusion are two distinct processes that mediate the mitochondrial dynamic morphology and integrity [126]. In healthy neurons, mitochondria need to maintain the fusion and fission mechanism in a balanced way for the normal functioning of the synapses [125]. The enzymes involved in the fusion process are mitofusion 1 (Mfn1), mitofusion 2 (Mfn2), and optic atrophy protein 1 (OPA1). The proteins involved in the fission process are dynamin-related protein-1 (Drp1) and fission 1 (Fis1). Mitochondrial biogenesis is the cellular process of producing new mitochondria and increasing the mitochondrial mass [127]. The regulation of biogenesis is modulated by nuclear factors including a set of nuclear transcription factors 1 and 2 (NRF 1 and NRF2), which control the cytochrome c and COX gene expression [128]. Any dysregulation in the mitochondrial dynamics and biogenesis might lead to excessive mitochondrial fragmentation and altered mitochondrial function resulting in mitochondrial dysfunction and eventually contribute to AD progression [129,130].

In astrocytes, impaired respiration rate and increased mitochondrial fragmentation (Drp1) have been reported upon IL-1*β* exposure [131]. In 3T3L1 adipocytes, TNF-*α* treatment alters mitochondrial morphology [132], along with smaller condensed mitochondria attributed by an increased level of Fis1 and decreased level of mitochondrial OPA1 [133,134]. In another in-vivo study, IL-6 downregulated Mfn1/Mfn2 and Fis1, which suggests neurotoxic effects of IL-6 [135]. In HFD mice, a decreased level of mitochondrial fusion genes (OPA1, Mfn1) and elevated expression of fission genes (Fis1, Drp1) along with increased pro-inflammatory cytokines has been reported [136]. Perturbation in mitochondrial function and release of mitochondrial contents in extracellular milieu activates the innate immune system, which can exacerbate the inflammatory response and alter the mitochondrial function. The production of ROS can provoke inflammation, and further mitochondrial dysfunction can lead to the production of pro-inflammatory IL-1*β* [137]. These studies suggested that inflammation and mitochondrial dysfunction operate in a synergic autotoxic feedback loop.

### 5.3. Inflammation and Mitochondrial Oxidative Stress

As a major source of ROS, mitochondria regulate the oxidative stress process [138]. Oxidative stress results from the imbalance between ROS production and detoxification in biological systems. ROS production is an important physiological by-product of the ETC. In the respiratory chain, while transferring the electron to molecular oxygen, about 0.4% to 5% of electrons lose their way and generate superoxide radicals (O_2_•^−^), which, in turn, activates the mitochondrial permeability transition pore and results in apoptosis [139]. The brain is a highly susceptible organ to oxidative stress when compared to other organs due to the high demand for energy and oxygen, high levels of peroxidizable polyunsaturated fatty acids, the relative paucity of antioxidants, and anti-oxidant defense mechanisms and relative abundance of potent ROS catalyst iron [140]. During obesity and HFD consumption, adipose tissue propagates the inflammation and secretes pro-inflammatory cytokines including TNF-*α*, IL-6, and IL-1*β*, which also induce ROS production [141]. The activation of these cytokines promotes nitric oxide and ROS generation by macrophages and monocytes. HFD consumption propagates the lipid peroxidation in the brain by elevated levels of ROS via a similar mechanism that has been found in the non-neuronal tissue [142]. In mice, upon HFD consumption, elevated expression of ERK, and inducible nitric oxide synthase (i-NOS) are associated with oxidative stress [143,144]. The occurrence of oxidative stress has been shown as one of the early events in AD development and plays a major role in the pathogenesis of AD [118,145,146], whereas oxidative damage has been estimated to occur before other prognoses including the onset of A*β* aggregates, tau pathologies, or inflammation in the AD brain [147,148]. The activation of these cytokines promotes nitrogen and ROS generation by macrophages and monocytes. It has also been shown that oxidative stress is related to the hippocampal dysfunction in obese mice [146,149]. In the AD brain, excessive levels of ROS production indicated by the presence of an increased level of oxidative stress markers including oxidized lipid, protein, and DNA has been noticed [118,146].

A*β* generates hydrogen peroxide (H_2_O_2_) by reducing metal ions and increasing the free radical production by zinc, iron, and copper, which is highly concentrated in the core and periphery of A*β* deposits [150]. A*β* plaques can lead to a cytosolic calcium ions overload by depleting Ca^2+^ storage in the endoplasmic reticulum (ER). Increased Ca^2+^ leads to decreased endogenous glutathione (GSH) levels and increased accumulation of ROS inside the cell [151]. ROS potentially can mediate the JNK/stress-activated protein kinase pathways that can be associated with tau hyper-phosphorylation [152]. A*β* can also initiate free radical formation upon Nicotinamide adenine dinucleotide phosphate hydrogen (NADPH) oxidase activation, which leads to over-accumulation of ROS. The A*β*-induced ROS can initiate tau hyper-phosphorylation and modify cellular signaling via p38 mitogen-activated protein kinase (MAPK) activation [153]. A*β* can generate free radicals when interacting with metal ions. Cu^2+^/Zn^2+^-bound A*β* has been found to possess a similar structure to the antioxidant superoxide dismutase (SOD) [154].

**Figure 1:**
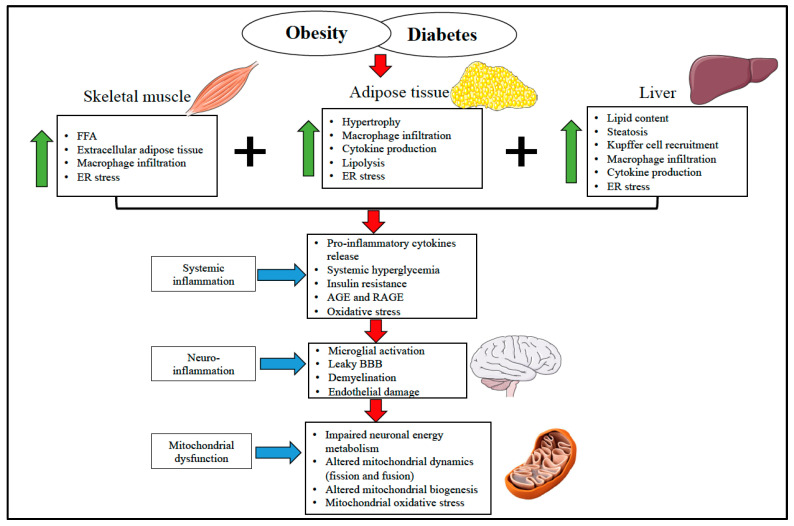
Overview of the diverse mechanism involved in obesity and diabetes-induced systemic and neuro-inflammation in mitochondrial and brain health. Skeletal muscle, adipose tissue, and liver are involved in glucose and lipid metabolism and play a vital role in developing adipose tissue dysfunction and insulin resistance during the obese condition. In obesity and diabetes, elevated free fatty acid, increased secretion of pro-inflammatory cytokines and macrophage infiltration, AGE and RAGE generation, and ER stress induce systemic inflammation. Systemic inflammation progressively initiates microglial activation, endothelial damage, and BBB disruption, which eventually leads to neuro-inflammation. These unfavorable inflammatory events alter the mitochondrial energy metabolism, mitochondrial dynamics, and biogenesis to escalate the mitochondrial oxidative stress collectively and trigger the mitochondrial assault. This gradually results in synapsis loss and neuronal death. FFA—Free Fatty Acid. ER—Endoplasmic Reticulum. AGE—Advanced Glycation End Products. RAGE—pattern recognition receptors of AGE. BBB—Blood-Brain Barrier.

Figure 1 provides an overview of how obesity and diabetes associated with elevated free fatty acids and impaired endocrine function leads to insulin resistance and systemic inflammation. This systemic inflammation triggers neuro-inflammation, which eventually hinders the metabolic and regulatory function of the brain mitochondria and leads to neuronal damage.

Collectively, it has been proposed that the development of AD in the brain is the consequence of increased oxidative stress derived from several mechanisms: (a) elevated lipid peroxidation, (b) increased protein and DNA oxidation, (c) reduced energy metabolism and activity of COX, (d) ability of A*β* to generate free radicals in the form of increased accumulation of some metal ions like mercury, iron, and aluminum, which can stimulate free radical formation by the Fenton and Haber-Weiss pathway, and increased advanced glycation end products, SOD-1, and malonaldehyde in NFT and senile plaques [155].

### 5.4. Inflammation and Cognitive Impairment

The association between inflammation and cognitive decline has been reported in several cross-sectional studies [156,157,158]. However, the data were limited by the number of subjects [156,157,158], lack of adequate follow-up duration [156], or enough inflammatory markers [156] or subjects who were more than 80 years old. Elevated IL-6 in midlife is associated with cognitive decline whereas increased CRP levels did not predict the concurrent cognitive decline [159]. One neuroimaging study found that IL-6 is consistently associated with cognitive decline. In a follow-up study, increased circulating IL-6 level was found to be associated with accelerated cognitive decline after a 10-year follow up [160]. In another study, IL-6 was found strongly associated with the higher volume of white matter hyper-intensities, decreased gray matter, and reduced hippocampal volume whereas, in the case of CRP, the association was weaker than IL-6. This suggests that an inflammatory process could be associated with alteration of the brain [161]. Animal studies indicate declined cognition associated with elevated inflammation and A*β* deposition [162]. However, some studies found a minor association between cognition and inflammation [156,157,158,163]. In the Rotterdam Study, elevated levels of pro-inflammatory cytokines including IL-6 and CRP were found to be associated with cognitive decline and executive function. In the Leiden 85-plus study, a systematic level of IL-6 was found to be associated with declined cognition and memory function after adjustment for Apolipoprotein E ɛ4 (APOE ɛ4) carriers [164]. On the other hand, the Amsterdam Longitudinal Aging Study did not find any association between inflammation and cognitive decline after adjusting for APOE ɛ4 [157]. Furthermore, cerebral small vessel disease (SVD) refers to intracranial vascular disease associated with clinical manifestation and neuroimaging features caused by any changes in the morphology of cerebral vessels, which are crucial for adequate cerebral blood flow and brain parenchyma [165]. SVD causes decreased cerebral blood flow, increased BBB permeability, impaired cerebral autoregulation, brain functionality loss, and cognitive decline in the elderly [165]. SVD has often detected the parenchymal alteration based on four different features in magnetic resonance imaging (MRI), which include white matter hyperintensities, lacunes, cerebral microbleeds, and enlarged perivascular spaces [166,167,168]. A detailed systematic review suggested a robust association of inflammation with SVD represented by the presence of increased vascular inflammatory markers, especially among patients with stroke, which indicates an alteration in the endothelium and BBB [169]. Mounting evidence also indicates the potential role of inflammation and cerebral SVD in neurodegeneration and related disorders in recent years [168,170].

## 6. Conclusions and Future Direction

In this review, we discussed how obesity and diabetes can lead to systemic and neuro-inflammation, and whether inflammation affects the overall mitochondrial functioning, dynamics, oxidative stress, and cognitive decline in patients with AD. After cautiously considering the facts, we can suggest (but not conclude) that obesity or diabetes-induced inflammation is associated with impaired mitochondrial health. However, the question remains regarding to what extent the systemic or neuro-inflammation directly affect the brain mitochondrial health and subsequent AD pathogenesis. The association between inflammation and cognitive decline could be the consequence of underlying disease conditions triggered by diabetes, obesity, or cardiovascular disease and their consequent comorbidities. If the answer to this question can be addressed, it would be easier to decide whether targeting anti-inflammatory agents alone will be an effective and valid approach to combat AD. It seems even an anti-inflammatory approach is not the central focus for fighting AD but will support the mainstream treatment options including inhibiting A*β* production and tau hyper-phosphorylation. If the anti-inflammatory approach effectively improves the systemic neuro-inflammation, which is one of the events in AD that happens before memory loss, it is possible to improve the progressive A*β* deposition and tau hyper-phosphorylation. However, it is impossible to differentiate the effects of inflammation from other pathologies that occur concomitantly. One of the known modulators for the immune system is diet [171]. Diet high in sugar and saturated fat (SFA) is associated with cognitive impairment especially in learning and memory function both in humans and animal models, independent of obesity or associated metabolic changes [172,173,174,175]. High consumption of sugar and SFA results in substantial memory deficits in animals and humans, however, the impact of relative consumption remains unclear due to variability between human consumption and the controlled diet feeding in animal studies [176,177,178]. The ketogenic diet could be one way to counteract the inflammation and improve mitochondrial biogenesis [179]. The ketogenic diet was tested on subjects with a mild cognitive impairment syndrome and found to be associated with reduced inflammation, enhanced energy metabolism, and potentially improved neurocognitive function [180]. Dietary administration of a medium-chain triglyceride supplement was found to improve cognitive performance [181,182]. The ketogenic diet has been reported to be helpful in reducing the frequency of epilepsy in children [183]. Similar findings have been reported in animal studies where mice fed a ketogenic diet improved neuro-inflammation represented by reduced brain mRNA levels of TNFα, IL-6, and IL-1β and increased the PGC1β mRNA levels indicating ketone bodies related changes [184,185]. Moreover, the Mediterranean diet (MD) has been used as a potential dietary intervention to combat AD-related dementia due to its anti-inflammatory potential [186,187]. Numerous neuroimaging studies reported that the MD has protective effects on the neuronal structure and AD-related morphological changes [188,189,190,191,192].

Notably, several epidemiological studies effectively demonstrate the protective effects of NSAIDs in combating AD via its anti-inflammatory actions along with A*β* lowering properties whereas clinical studies did not support such an improvement [193,194,195,196,197]. More research is needed to conclude if obesity and diabetes associated with chronic inflammation and induced mitochondrial dysfunction can be identified as a biomarker that affects AD pathogenesis and whether the anti-inflammatory approach is a suitable therapeutic option to combat AD.
